# Frequency of Insulin Resistance in Overweight Patients With Metabolic‐Associated Fatty Liver Disease (MAFLD) at a Tertiary Healthcare Centre in Bangladesh

**DOI:** 10.1002/edm2.70271

**Published:** 2026-07-01

**Authors:** Md. Mohiuddin Rozaik, Md. Kawsar Uddin, Syed Azmal Mahmood, Sadia Islam Sukonna, Tasnim Nafian, Kazi Ali Aftab, Khaled Mahbub Murshed, Md Abul Kalam Azad

**Affiliations:** ^1^ Department of Internal Medicine Bangladesh Medical University Dhaka Bangladesh; ^2^ Department of Gastroenterology Dhaka Medical College & Hospital Dhaka Bangladesh; ^3^ Department of Endocrinology Dhaka Medical College Hospital Dhaka Bangladesh; ^4^ Department of Dermatology & Venerology Bangladesh Medical University Dhaka Bangladesh

**Keywords:** acanthosis nigricans, HOMA‐IR, insulin resistance, MAFLD

## Abstract

**Background:**

In Bangladesh, the prevalence of MAFLD patients is about 33.86%. Most of the patients with MAFLD are also strongly associated with hepatic and adipose tissue insulin resistance (IR). Homeostasis model assessment of insulin resistance (HOMA‐IR) is a simple and useful method for evaluating insulin resistance and β cell function.

**Methods:**

This observational cross‐sectional study was carried out in the outpatient department of Hepatology, Bangladesh Medical University. The subjects were non‐alcoholic overweight patients with sonographic evidence of fatty liver disease aged 18 years or above. Participants were subjected to a pre‐designed questionnaire including personal data. Assessment of medical history, physical examinations and investigations to measure IR were performed. Statistical analyses were carried out by using the SPSS version PC 26.0.

**Results:**

The Majority of the participants, precisely 60% (95% confidence interval [CI]: 45.2, 73.6) had insulin resistance (IR) and median HOMA‐IR index was 2.85. Patients with acanthosis nigricans had significantly higher IR biochemically (Mean HOMA‐IR index; 5.79 vs. 2.89), which was significant statistically. No significant correlation was observed between HOMA‐IR and alternative metabolic markers, including METS‐IR, TG/HDL‐c ratio and BMI.

**Conclusion:**

A high frequency (60%) of insulin resistance was found among overweight, non‐diabetic patients with MAFLD. This finding underscores the immense value of regular screening for hyperglycemia in this population. With the increasing burden of metabolic syndrome, early identification of IR would enable timely preventive interventions—potentially slowing the progression to type 2 diabetes and consequently reducing subsequent risk of developing diabetes‐related complications.

## Graphical abstract

Among overweight, non‐diabetic MAFLD patients at a Bangladeshi tertiary centre, 60% had insulin resistance (median HOMA‐IR: 2.85). Acanthosis nigricans significantly correlated with higher HOMA‐IR (5.79 vs. 2.89). No significant correlation existed between HOMA‐IR and BMI, METS‐IR, or TG/HDL‐c ratio. Routine hyperglycemia screening is essential to prevent progression to type 2 diabetes as well as MAFLD.

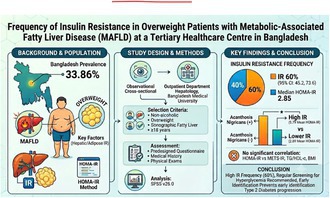

## 1 Introduction

MAFLD affects roughly a quarter of adults worldwide and is tightly linked to obesity, type 2 diabetes and systemic insulin resistance (IR) [1, 2, 3, 4, 5, 6, 7]. It represents a wide spectrum of liver damage, ranging from pure steatosis to non‐alcoholic steatohepatitis (NASH) and eventually to cryptogenic cirrhosis resulting in liver failure [8]. IR is a pathophysiologic hallmark across the MAFLD spectrum, encompassing hepatic and adipose tissue insulin insensitivity affecting hepatic glucose production (HGP), glucose disposal, lipolysis and lipid oxidation along with reduced whole‐body insulin action [5, 6, 9]. While clamp studies are the gold standard, HOMA‐IR offers a practical surrogate in clinical and epidemiologic settings [10, 11]. However, values used for this parameter have shown large variability; values above or equal to 2.0 or 2.5 show enhanced diagnostic value in distinguishing non‐alcoholic fatty liver disease carriers from control group individuals [12].

South Asian populations carry a disproportionate MAFLD/IR burden, with regional data suggesting high prevalence and distinctive risk phenotypes [2, 3, 13]. Bangladesh‐specific evidence indicates substantial MAFLD prevalence (33.86%) and associated metabolic risk [13]. We aimed to quantify IR frequency and assess clinical correlates among overweight adults with MAFLD in a tertiary outpatient setting in Bangladesh.

## 2 Materials and Methods

### 2.1 Study Design, Site and Sampling

This observational cross‐sectional study was conducted among a total of 50 overweight (BMI: > 23 kg/m^2^; according to WHO) adult (≥ 18 years of age) sonographically diagnosed cases of fatty liver disease (FLD) [14]. Participants were recruited from the patients attending the outpatient facility of the department of Hepatology of BMU (Outpatient Facility Building no. 1, Room no 405,406) dating from February 2022 to October 2022 using consecutive sampling.

### 2.2 Exclusion Criteria

Those who were alcoholic (≥ 20 g/day) or had T2DM/IGT (by performing FBS and 75 g OGTT [15]) or previously diagnosed chronic hepatic illnesses (Viral hepatitis, Wilson's disease, autoimmune hepatitis, Cholestatic liver disease, Hemochromatosis, portal HTN) were excluded from the study.

### 2.3 Ethical Consideration

The study protocol was approved by the Institutional Review Board of BMU (No: 3492) and conforms to the ethical guidelines of the 1995 declaration of Helsinki.

### 2.4 Data Collection

Data were collected using a pre‐designed structured questionnaire. Relevant demographic, clinical and medical features such as age, sex, socio‐economic status, food habit, working status, smoking habit, history of diabetes, use of medications and history of serious co‐morbid diseases were noted from each participant.

Anthropometric measurements were performed using standardized techniques. Height was measured without shoes using a stadiometer and recorded to the nearest 0.5 cm. Weight was measured with the participants wearing light clothing using a calibrated weighing scale and recorded to the nearest 0.1 kg. Body mass index (BMI) was calculated as weight in kilograms divided by the square of height in meters (kg/m^2^).

Clinical examination was performed to identify signs suggestive of insulin resistance, including increased waist circumference and the presence of acanthosis nigricans [16].

### 2.5 Sample Collection and Analysis

Blood samples were collected from all participants after an overnight fasting period of at least 8 h and 2 h after 75 g oral glucose intake. Venous blood samples were drawn under aseptic conditions and then processed for laboratory investigations.

Fasting plasma glucose, fasting insulin levels and OGTT were measured using the *SIEMENS Atellica Solution* (Immunoassay & Clinical Chemistry Analyser). These measurements were used to assess insulin resistance.

The presence and grading of fatty infiltration of the liver is recorded with abdominal ultrasonography because of its availability and cost effectiveness compared to the gold standard Liver Biopsy. The diagnosis and grading of MAFLD was based on internationally accepted USG criteria: (i) degree of increased hepatic echogenicity relative to the right kidney; (ii) presence and depth of posterior acoustic enhancement and (iii) blurring or loss of definition of the portal vein walls due to increased parenchymal echogenicity [17].

To assess MAFLD, the *Philips Affiniti 30* Ultrasonography machine was used by an expert radiologist.

### 2.6 Biomarker and Indices

Insulin resistance was assessed using the Homeostasis Model Assessment of Insulin Resistance (HOMA‐IR). It is one of the most widely used indices based on fasting parameters. It is considered to be a reliable surrogate measure of in vivo insulin sensitivity in humans. The HOMA‐IR index was calculated using fasting plasma glucose and fasting insulin values according to the following formula [18]:

$$\begin{array}{c} \text{HOMA}-\mathrm{IR}=\left\{\left[\text{fasting insulin}\;\left(\mathrm{\mu U}/\mathrm{mL}\right)\right]\right.\\ \;\left.\times \left[\text{fasting glucose}\;\left(\text{mmol}/L\right)\right]\right\}/22.5 \end{array}$$

A person with HOMA‐IR levels ≥ 2.5 was considered to be insulin resistant. This threshold is widely accepted for adult metabolic studies, with validation against the hyperinsulinemic‐euglycemic clamp in diverse populations, adoption in South Asian research and support from a Bangladeshi normoglycemic cohort where the 75th percentile HOMA‐IR was 2.6 [18, 19, 20].

According to the revised NCEP ATP III criterion for the classification of MetS, study participants should have at least three of the five following components: WC (> 90 cm for males and > 80 cm for females); BP (≥ 130/85 mm/Hg or use of anti‐hypertensive drugs); HDL (< 40 mg/dL for males and < 50 mg/dL for females or use of antilipidemic drugs); TG (≥ 150 mg/dL or use of dyslipidemia drugs); and/or FBS (> 100 mg/dL or use of hypoglycaemic drugs) [21].

The METS‐IR, as a surrogate marker to evaluate insulin sensitivity is calculated using the following formula: [22]

$$\text{METS}-\mathrm{IR}=\left[\ln\left\{\left(2\times \mathrm{FBS}\right)+\mathrm{TG}\right\}\times \mathrm{BMI}\right]/\ln\left(\mathrm{HDL}-c\right)$$

where FBS is fasting blood sugar in mg/dL, TG is fasting triglycerides in mg/dL, HDL‐c is high‐density Lipoprotein cholesterol in mg/dL and BMI is body mass index in kg/m^2^.

### 2.7 Data Analysis

After collection of all the required data, these were checked, verified for consistency and tabulated using the SPSS/PC 26.0 software.

Continuous variables were summarized as mean ± standard deviation or median with interquartile range, whereas categorical variables were presented as frequencies and percentages.

The primary outcome—the frequency of IR—was defined as a binary variable applying the validated HOMA‐IR ≥ 2.5 cut‐off.

As a secondary analysis, the association between body mass index (BMI) and HOMA‐IR as a continuous variable was examined. Because HOMA‐IR values were markedly right‐skewed, the relationship was assessed first by Spearman's rank correlation. As a supplementary check, HOMA‐IR was log‐transformed and then linear regression was performed.

Group comparisons were performed using Student's *t*‐test, Pearson's chi‐square test and Fisher's exact test, as appropriate.

A *p*‐value of less than 0.05 was considered statistically significant at a 95% confidence level.

## 3 Results

### 3.1 The Study Population

Total 50 participants with fatty liver disease confirmed by ultrasonography were included in this study. The largest proportion of participants belonged to the 35–49 years age group (58%), followed by those aged 20–34 years (36%), while only 6% were between 50 and 60 years of age.

In terms of gender distribution, 58% were female whereas 42% of the study population were male. Most participants were Muslim (94%), reflecting the religious distribution of the population in Bangladesh.

Regarding marital status, 84% of participants were married, while 8% were unmarried and another 8% were widowed.

In terms of residence, 56% of participants lived in urban areas, whereas 44% resided in rural areas.

Among the participants, homemakers formed the largest group (52%), followed by non‐government employees (32%). Smaller proportions included government employees (4%), businessmen (2%), unemployed individuals (2%) and other occupations (8%).

Analysis of educational status revealed that 40% of participants had education below the SSC level, whereas 26% had completed graduation or higher education.

Regarding socioeconomic status, the majority of participants belonged to the upper‐middle income category (48%), followed by high‐income (24%), lower‐middle income (18%) and low‐income groups (10%).

Overall, the study population consisted predominantly of middle‐aged, urban residents who were married, with a higher proportion of female participants (Table 1).

**TABLE 1:** Socio‐demographic characteristics of the participants (*n* = 50).

Characteristics	Number	Percentage
Gender		
Male	21	42%
Female	29	58%
Age in years		
20–34	18	36%
35–49	29	58%
50–60	3	6%
Religion		
Islam	47	94%
Hindu	3	6%
Marital status		
Unmarried	4	8%
Married	42	84%
Widow	4	8%
Residence		
Rural	22	44%
Urban	28	56%
Occupation		
Government employee	2	4%
Non‐government employee	16	32%
Businessman	1	2%
Homemaker	26	52%
Unemployed	1	2%
Others	4	8%
Education		
Illiterate	5	10%
Below SSC	20	40%
SSC	5	10%
HSC	7	14%
Graduation and above	13	26%
Income		
Low	5	10%
Low middle	9	18%
Upper middle	24	48%
High	12	24%

### 3.2 Clinical and Anthropometric Parameters as Indicators of Insulin Resistance

BMI, waist circumference and presence of acanthosis nigricans were used as parameters for analysis (Table 2).

**TABLE 2:** Clinical and anthropometric parameters as indicators of insulin resistance.

Clinical variable	Category	Insulin		Total (*n*)	*p*
		Sensitive (*n*)	Resistant (*n*)		
Body mass index (BMI)	Overweight^a^	3	5	8	1.00^d^
	Obese^b^	17	25	42	
Waist circumference	Normal	14	14	28	0.103^e^
	Increased^c^	6	16	22	
Acanthosis nigricans	Absent	20	24	44	0.037^d^
	Present	0	6	6	

^a^ Overweight defines as BMI is 23–25 kg/m^2^.

^b^ Obese defined as BMI > 25 kg/m^2^.

^c^ Waist circumference increased defined as > 40 in. for men/> 35 inch for women.

^d^ *p*‐value measured by Fisher's Exact test.

^e^ *p*‐value measured by Chi‐square test.

Among the 50 study participants, 60% (*n* = 30) were found to be biochemically insulin resistant, while 40% (*n* = 20) were insulin sensitive based on the HOMA‐IR cutoff value.

**FIGURE 1:**
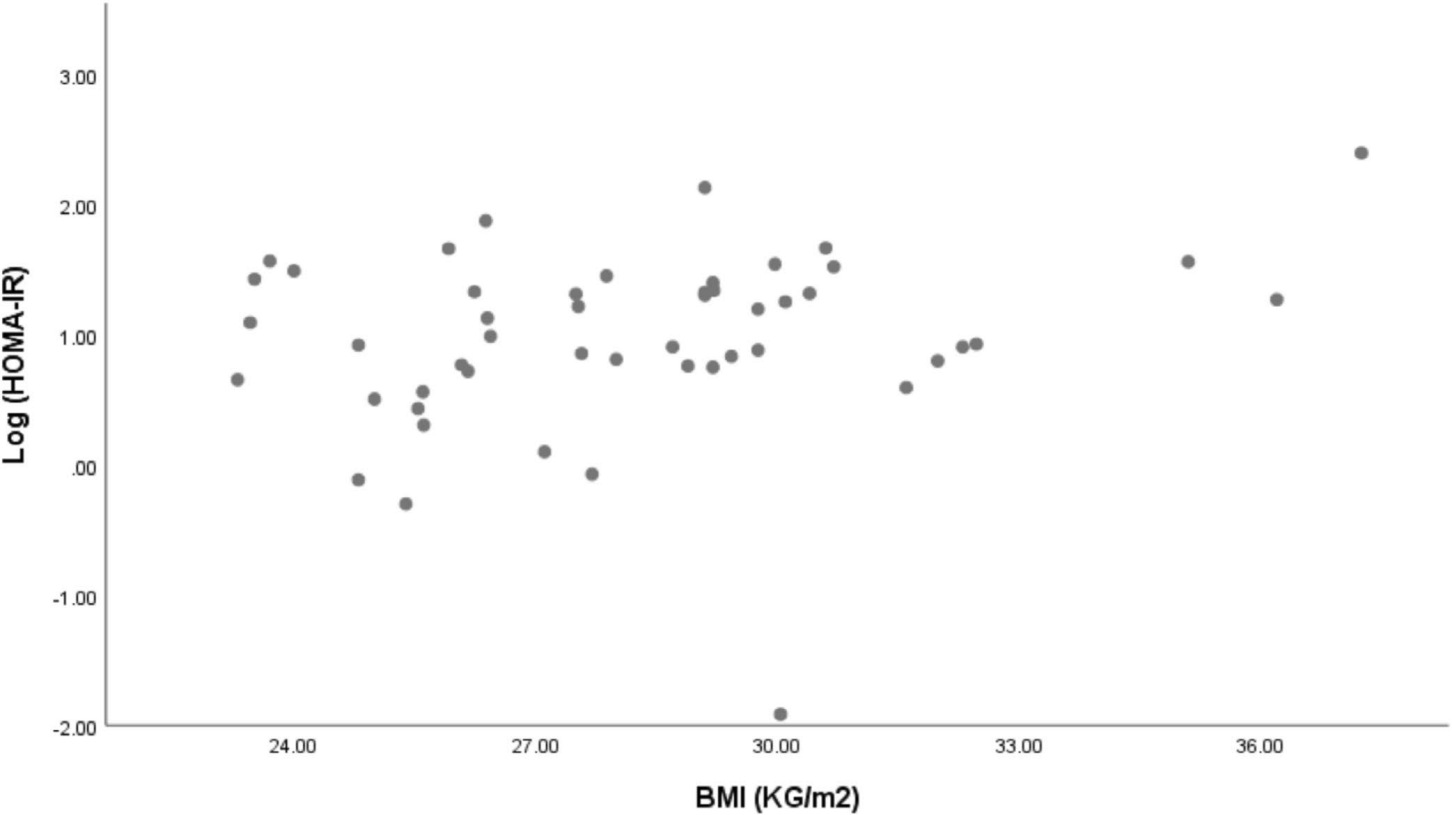
Scatterplot showing the relationship between log‐transformed HOMA‐IR and BMI (kg/m^2^).

Among all participants, 16% (*n* = 8) patients were overweight and 84% (*n* = 42) patients were obese. The distribution of HOMA‐IR values was markedly right‐skewed (skewness = 1.739). Therefore, a Spearman's correlation was used as the primary analysis of the association between BMI and HOMA‐IR. This showed a weak positive relationship that was not statistically significant (*r*_*s*_ = 0.239, *p* = 0.095). As a supplementary check, HOMA‐IR was log‐transformed and then linear regression on BMI was performed (Figure 1). BMI was also not a significant predictor (*β* = 0.225, *R* ^2^ = 0.051, *p* = 0.117).

Waist circumference was also evaluated as a clinical indicator of insulin resistance. Among participants with increased waist circumference, 72.7% were insulin resistant, whereas 50% of participants with normal waist circumference were insulin resistant. However, this association also did not reach statistical significance (*p* = 0.103).

The presence of acanthosis nigricans showed a strong association with insulin resistance. All participants with acanthosis nigricans (100%) were found to be insulin resistant biochemically. This relationship was statistically significant (*p* = 0.037), suggesting that acanthosis nigricans may serve as an important clinical marker of insulin resistance in patients with fatty liver disease.

### 3.3 Comorbidity and Insulin Resistance

HOMA‐IR values were higher in participants with hypertension (4.17 ± 3.01, *n* = 11) and hypothyroidism (4.19 ± 3.36, *n* = 2) compared to those without any comorbidity (2.93 ± 1.36, *n* = 34). However, these differences were not statistically significant (*F*[5, 44] = 0.973, *p* = 0.445) (Table 3).

**TABLE 3:** Comorbidity and insulin resistance.

Comorbidity	*N*	HOMA‐IR (mean ± SD)	*p*
No comorbidity	34	2.93 ± 1.36	0.445
Hypertension (HTN)	11	4.17 ± 3.01	
Hypothyroidism	2	4.19 ± 3.36	
Other categories^a^	3	2.65	
Total	50	3.24	

^a^ Other categories include subclinical thyrotoxicosis and PCOS.

### 3.4 TG/HDL‐c Ratio and Insulin Resistance

Mean TG/HDL‐c ratio of all participants was 5.73 (±3). Median TG/HDL‐c ratio was 4.97. Among the participants who had TG/HDL‐c ratio less than 5 (*n* = 25), 52% (*n* = 13) were insulin resistant; in contrast, 67% (*n* = 16) of participants having TG/HDL‐c ratio equal to or higher than 5 (*n* = 24) were insulin resistant.

**FIGURE 2:**
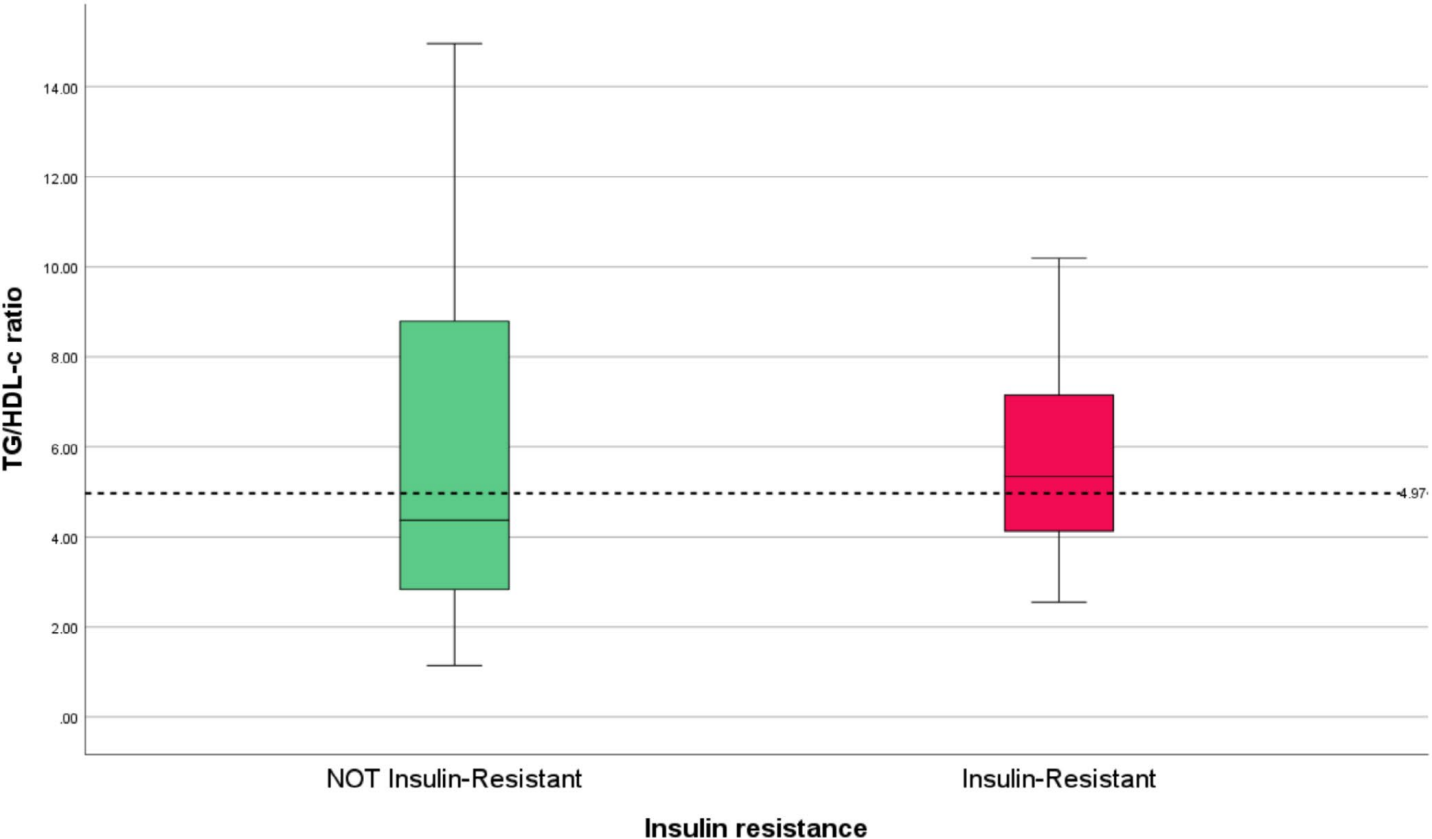
Boxplot showing TG/HDL‐c ratio in insulin‐resistant and insulin‐sensitive patients.

In insulin‐sensitive patients the median TG/HDL‐c ratio was 4.37. The 25% and 75% interquartile were 2.8 and 8.8 respectively. On the other hand, the median TG/HDL‐c ratio of insulin resistant patients was 5.36. The 25% and 75% interquartile were 4.1 and 7.2 respectively. (Figure 2). To determine the relationship between the TG/HDL‐c ratio and insulin resistance, a Spearman's correlation was conducted. In our study, no statistically significant correlation was found (*r*_*s*_ = 0.096, *p* = 0.513).

### 3.5 Sonographic Grading of MAFLD and Insulin Resistance

By using the Kruskal Wallis test, it was found that there was no relationship between Insulin resistance and the grading of MAFLD, with no statistically significant result (*p* value 0.817) (Table 4). The median HOMA‐IR index was 2.998 in MAFLD grade I, 2.795 in MAFLD grade II and 2.31 in NAFLD grade III. These findings suggest that the degree of hepatic steatosis detected on ultrasonography may not necessarily correlate with the level of insulin resistance.

**TABLE 4:** Sonographic grading of fatty liver disease and HOMA‐IR index.

FLD grading	HOMA‐IR (median)	*p*
NAFLD Grade I	2.998	0.817*
NAFLD Grade II	2.795	
NAFLD Grade III	2.31	

* *p*‐value measured by Kruskal–Wallis test (ns = not significant).

### 3.6 Metabolic Syndrome and Insulin Resistance

Metabolic syndrome was present in 53.2% (*n* = 25/47) of the valid cases. Females had significantly higher prevalence than males (74.1% vs. 25.0%; *p* = 0.001). Insulin‐resistant participants also showed a significantly higher prevalence than non‐resistant individuals (69.0% vs. 27.8%; *p* = 0.006) (Table 5).

**TABLE 5:** Metabolic syndrome and HOMA‐IR index.

Metabolic syndrome	*N*	Insulin		*p*
		Sensitive (*n*)	Resistant (*n*)	
Absent	22	13	9	0.006
Present	25	5	20	
Total	47^a^			

^a^ *N* = 47 for MetS due to missing laboratory data.

### 3.7 METS‐IR and Insulin Resistance

**FIGURE 3:**
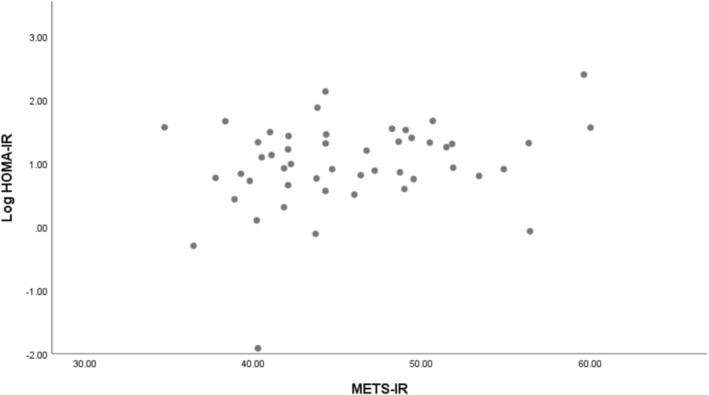
Scatterplot showing the relationship between log‐transformed HOMA‐IR and METS‐IR.

A simple linear regression showed the METS‐IR index was a marginally non‐significant predictor of log‐transformed HOMA‐IR, accounting for 7.2% of the variance (*R* ^2^ = 0.072, *p* = 0.062) (Figure 3).

## 4 Discussion

The majority of the participants, precisely 60% (95% confidence interval [CI]: 45.2, 73.6) had insulin resistance and median HOMA‐IR index was 2.85. Comparable two studies done by Sejooti et al. and Parvin et al. on Bangladeshi population reported IR prevalence of 60.2% (HOMA‐IR ≥ 2.8) and 78.8% (HOMA‐IR ≥ 3) in obese patients respectively [23, 24]. But neither of them included MAFLD in the inclusion criteria. In a study done by Singh et al. which was conducted in Coastal Eastern India, similar results were observed (IR 60%, HOMA‐IR index 2.6) [25]. Another study in India was done by Suresh et al. which had comparable results; where among participants with MAFLD, 66% were insulin resistant [26]. Two more studies which monitored IR among MAFLD/Obese patients in Indian population by Mathew et al. [27] and Bhat et al. [28] found that the prevalence of IR were 76.7% (HOMA‐IR ≥ 3) and 97.5% (HOMA‐IR > 1.64). In our study, female patients were marginally more insulin resistant than male patients (62.07% vs. 57.14%); which isn't statistically significant (*p* = 0.726).

Due to paucity of researches in this specific sector in Bangladesh, only one study was found done by Israt et al. which had evaluated association between MFALD and IR. But all study participants had impaired glucose tolerance and they were included irrespective of their BMI which substantially differs from our study. 57.1% (20 out of 35 patients) of their MFALD patients had IR [29].

Mean HOMA‐IR index across the participants was 3.24 ± 1.9 which is significantly higher than what was found (mean HOMA‐IR index 1.66) in one large study (done by Bhowmik et al. in 2009) [30] which aimed to identify IR in the general Bangladeshi population with normal blood glucose, which was statistically significant (*p* < 0.001).

In this study, most of the participants were of the 35–49 years age group (58%) and the majority were Muslim (94%) as well as married (84%). Prevalence of insulin resistance slightly increased with age (not statistically significant, *p* = 0.880) in this study, which is comparable to the study done by Gayoso‐Diz et al. [31]; where HOMA‐IR level was higher in women aged above 50 years. On the contrary, according to the other two studies conducted by Karakelides et al. [32] and Thanikachalam et al. [33] age had no independent effect on insulin sensitivity. Interestingly, almost 90% of female patients were homemakers in occupation, and 62.07% of them were insulin resistant. In this study, frequency IR was higher in urban participants than rural participants (64.3% vs. 54.55%), which was not statistically significant (*p =* 0.485). Median HOMA‐IR index was 3.06 and 2.52 respectively. This result is similar to the study of Thanikachalam et al., which was also done in south Asian population [33].

The mean BMI among all participants was 28.2 (±3.17). According to WHO Asian‐BMI classification, 84% of the study participants were obese. BMI could not predict IR in our study. When our patients were categorized in two groups based on WHO BMI classification, there wasn't any statistically significant difference in IR between overweight and obese patients (62.5% vs. 59.5%; *p* = 0.875).

Patients who had acanthosis nigricans (*n* = 6), all of them had IR biochemically; which was statistically significant (*p* < 0.05). Mean HOMA‐IR index in this group of patients was significantly higher than the mean of all other participants (5.79 vs. 2.89), which was significant statistically (*p* < 0.001). A cross‐sectional study done by Nithun et al. among the Indian young population found a similar disparity in IR index between patients with acanthosis nigricans and patients having no acanthosis nigricans (2.42 vs. 1.32) [34]. In all insulin resistant patients, only 20% (*n* = 6) patients had acanthosis nigricans, making it a very specific but poorly sensitive clinical tool to diagnose underlying insulin resistance. On the other hand, no statistically significant association (*p* = 0.103) was found between waist circumference and IR in this study. Among the participants fulfilling waist circumference criteria for IR (*n* = 22) only 72.27% patients were insulin resistant biochemically and half of the patients who did not cross the cutoff value of waist circumference for IR were found to be biochemically insulin resistant; making this clinical tool only 53.33% sensitive and 70% specific. According to a large retrospective study done by Wahrenberg et al. among white population (HOMA‐IR cutoff value 3.99) sensitivities and specificities were between 94%–98% and 61%–63% respectively which contradicts with what this study has found [35]. It is most likely due to sample size differences and limitation of resources of this study as well as large anthropometric and ethnic dissimilarities between these two studies.

HOMA‐IR values were elevated in participants with hypertension and hypothyroidism relative to those without comorbidities; however, this difference was not statistically significant (*p* = 0.445).

In this study, the mean TG/HDL‐c ratio was 5.73 ± 3, markedly exceeding the conventional cutoff value of 3. However, a Spearman's correlation revealed no significant association between TG/HDL‐c ratio and insulin resistance (IR). This contrasts with a large cross‐sectional study by Gong et al., conducted in an American population, which demonstrated a positive, nonlinear relationship between TG/HDL‐c ratio and IR [36].

No relationship between Insulin resistance and the sonographic grading of MAFLD was found in this study, as indicated by the lack of any statistically significant (*p* value > 0.05). This finding contradicts the results of a cross‐sectional study conducted by Marpaung et al. in Indonesia, which had reported a significant relationship between these variables [37]. The discrepancy may be attributed to differences in demographics between the study populations, the small sample size in our groups and the limited sensitivity of ultrasonography in accurately grading MAFLD.

The observed 53.2% prevalence of metabolic syndrome in our cohort is substantially higher than the 30% baseline average reported for the general population of Bangladesh. This discrepancy can be attributed to the participant selection criteria, which selectively enrolled patients who were both overweight and diagnosed with MAFLD [38].

METS‐IR showed only a marginal, non‐significant positive trend in predicting log‐HOMA‐IR within this specific overweight MAFLD cohort, which differs from a euglycemic‐hyperinsulinemic clamp validated study done by Bello‐Chavolla et al. [22].

## 5 Conclusion

This study confirms a high frequency (60%) of insulin resistance among overweight, non‐diabetic patients with MAFLD in a Bangladeshi tertiary care setting. This finding underscores the immense value of regular screening for hyperglycemia in this population. In the context of tripling global obesity since 1975 (according to WHO), the high prevalence rate of insulin resistance detected in our overweight MAFLD cohort heralds an increased burden of metabolic syndrome and diabetes mellitus in Bangladesh. Early identification of IR would enable timely preventive interventions—potentially slowing the progression to type 2 diabetes and consequently reducing subsequent risk of developing diabetes related complications, which are already overwhelming our healthcare facilities.

## Author Contributions

**Syed Azmal Mahmood:** methodology, visualization, writing – original draft, investigation. **Md. Kawsar Uddin:** conceptualization, methodology, writing – original draft, investigation, visualization. **Khaled Mahbub Murshed:** methodology, data curation, formal analysis, visualization, resources, writing – original draft. **Sadia Islam Sukonna:** writing – original draft, methodology, data curation, supervision. **Md Abul Kalam Azad:** conceptualization, methodology, data curation, validation, visualization, resources, writing – review and editing, writing – original draft. **Md. Mohiuddin Rozaik:** conceptualization, data curation, methodology, software, investigation, validation, formal analysis, supervision, visualization. **Tasnim Nafian:** methodology, software, data curation, investigation, writing – original draft, visualization. **Kazi Ali Aftab:** conceptualization, methodology, software, data curation, investigation, validation, formal analysis, supervision, visualization, project administration, resources, writing – original draft, writing – review and editing.

## Funding

The authors have nothing to report.

## Ethics Statement

The study protocol was approved by the Institutional Review Board of Bangladesh Medical University, Dhaka, Bangladesh (Registration No: 3492, Date: 28/10/2021) and conforms to the ethical guidelines of the 1995 declaration of Helsinki.

## Consent

Voluntary informed written consent was taken from each subject after a thorough explanation of the procedure and purpose of the study. Each participant enjoyed the right to participate or refuse and even withdraw from the study at any point in time without any compromise in their medical treatment. Data obtained from the participants was regarded as confidential and preserved by the investigator. Participants were given a unique identification number so that anonymity was maintained. Pertinent data were disclosed only to concerned supervisors during the processing of the data and preparation of the manuscript.

## Conflicts of Interest

The authors declare no conflicts of interest.

## Data Availability

The data that support the findings of this study are available on request from the corresponding author. The data are not publicly available due to privacy or ethical restrictions.
